# Swiss Survey on current practices and opinions on clinical constellations triggering the search for PNH clones

**DOI:** 10.3389/fmed.2023.1200431

**Published:** 2023-07-26

**Authors:** Alicia Rovó, Mathilde Gavillet, Beatrice Drexler, Peter Keller, Jenny Sarah Schneider, Giuseppe Colucci, Yan Beauverd, Hendrika Anette van Dorland, Matthias Pollak, Adrian Schmidt, Andrea De Gottardi, Marina Bissig, Thomas Lehmann, Michel A. Duchosal, Sacha Zeerleder

**Affiliations:** ^1^Department of Hematology and Central Hematology Laboratory, Inselspital, Bern University Hospital, Bern, Switzerland; ^2^Service and Central Laboratory of Hematology, Department of Oncology, Lausanne University Hospital (CHUV), Lausanne, Switzerland; ^3^Department of Laboratory Medicine and Pathology, Lausanne University Hospital (CHUV), Lausanne, Switzerland; ^4^Division of Hematology, University Hospital Basel, Basel, Switzerland; ^5^Hospital Langenthal, Langenthal, Switzerland; ^6^Clinica Sant'Anna, Lugano, Switzerland; ^7^Division of Hematology, Geneva University Hospitals and Faculty of Medicine, Geneva, Switzerland; ^8^Appletree CI Group AG, Winterthur, Switzerland; ^9^Department of Internal Medicine, Clinic of Medical Oncology and Hematology, Municipal Hospital Zurich Triemli, Zürich, Switzerland; ^10^Servizio di Gastroenterología e Epatologia, Ente Ospedaliero Cantonale, Università della Svizzera Italiana, Lugano, Switzerland; ^11^Department of Medical Oncology and Hematology, University Hospital of Zürich, Zürich, Switzerland; ^12^Kantonsspital St. Gallen, Clinic for Medical Oncology and Hematology, St. Gallen, Switzerland; ^13^Department of Hematology, Kantonsspital Luzern, Lucerne and University of Bern, Bern, Switzerland

**Keywords:** paroxysmal nocturnal hemoglobinuria (PNH), aplastic anemia, bone marrow failure (BMF), PNH clone, survey

## Abstract

This national survey investigated the current practice in Switzerland by collecting participants' opinions on paroxysmal nocturnal hemoglobinuria (PNH) clone assessment and clinical practice.

**Aim:**

This study aimed to investigate clinical indications prompting PNH clones' assessment and physician's accessibility of a flow cytometry facility, and also to understand clinical attitudes on the follow-up (FU) of patients with PNH clones.

**Methods:**

The survey includes 16 multiple-choice questions related to PNH and targets physicians with a definite level of experience in the topic using two screener questions. Opinion on clinical management was collected using hypothetical clinical situations. Each participant had the option of being contacted to further discuss the survey results. This was an online survey, and 264 physicians were contacted through email once a week for 5 weeks from September 2020.

**Results:**

In total, 64 physicians (24.2%) from 23 institutions participated (81.3% hematologists and 67.2% from university hospitals). All had access to flow cytometry for PNH clone testing, with 76.6% having access within their own institution. The main reasons to assess for PNH clones were unexplained thrombosis and/or hemolysis, and/or aplastic anemia (AA). Patients in FU for PNH clones were more likely to be aplastic anemia (AA) and symptomatic PNH. In total, 61% of the participants investigated PNH clones repetitively during FU in AA/myelodysplastic syndromes patients, even when there was no PNH clone found at diagnosis, and 75% of the participants tested at least once a year during FU. Opinions related to clinical management were scattered.

**Conclusion:**

The need to adhere to guidelines for the assessment, interpretation, and reporting of PNH clones emerges as the most important finding, as well as consensus for the management of less well-defined clinical situations. Even though there are several international guidelines, clear information addressing specific topics such as the type of anticoagulant to use and its duration, as well as the indication for treatment with complement inhibitors in some borderline situations are needed. The analysis and the discussion of this survey provide the basis for understanding the unmet needs of PNH clone assessment and clinical practice in Switzerland.

## Introduction

Paroxysmal nocturnal hemoglobinuria (PNH) is a rare chronic hematologic disease caused by uncontrolled terminal complement activation (1). Over the decades, the advent of highly sensitive flow cytometry techniques for the detection of PNH clones has led to the identification of a growing pool of patients with PNH clones (2, 3). PNH cells are characterized by the deficiency of glycosylphosphatidylinositol-anchored proteins (GPI-APs) on the cell surface due to an acquired mutation of the PIG-A gene in one or more hematopoietic stem cells (4). Both white blood cells and red blood cells (RBCs) should be evaluated to assess the size of the PNH clonal population. Testing of RBCs alone may underestimate the PNH population because of the short lifespan of the PNH red cells (5–7). Small to moderate PNH clones are frequently found in patients with acquired aplastic anemia (AA) (8, 9) and occasionally with myelodysplastic syndromes (MDSs) (10). PNH clones can also be found in patients with unexplained asymptomatic cytopenia (11, 12), thrombosis at unusual sites (13–15), pain associated with smooth muscle dystonia (16), and a variety of rare Coombs-negative hemolysis scenarios (17–20); however, it is important to mention that the traditional inclusion of PNH only in Coombs-negative hemolytic conditions should be reviewed (21). Furthermore, in bone marrow failure, the finding of a PNH clone can provide an important diagnostic clue about the immune-mediated pathogenesis of underlying marrow aplasia (22, 23). Thrombosis (arterial or venous) is the most harmful complication and represents the leading cause of death in the pre-treatment (with complement inhibitor) era (24). The underlying mechanisms associated with the risk of thrombosis are still poorly understood, and thus the prediction of PNH complications in such patients is limited (25–27). Furthermore, the clinical significance of PNH clones should be defined because, in most patients with PNH clones, these clones will be subclinical and their significance will become evident only during follow-up (28, 29).

A lack of general awareness of this rare disease as well as a lack of specific knowledge about its diagnosis, treatment, and management, even among hematologists or other medical specialties, likely leads to misdiagnosis and underestimation of PNH. One of the most important reasons for the underdiagnoses of PNH is the difficulty in accessing second-level flow cytometry facilities capable of performing the recommended 6- or 7-color diagnostic tests (30).

The primary aim of this study is to better understand the current practice in Switzerland for PNH clone assessment by collecting opinions on clinical presentations that should prompt PNH investigation. Furthermore, we aimed to identify physicians' access to clone detection methods for their patients and their attitudes toward monitoring patients harboring PNH clones. Finally, we gathered opinions on the clinical management of PNH.

## Materials and methods

### Study design

This study was a real-world, prospective, cross-sectional online survey conducted among physicians in Switzerland. An online questionnaire specifically designed for the present study by the two main investigators was used to collect data. The questionnaire consisted of 16 multiple-choice questions, including three hypothetical clinical situations (Supplementary material).

### Inclusion and exclusion criteria and participant recruitment

As this study did not involve the collection of health-related data of patients and all the collected data were anonymized, neither ethics approval nor written consent from the participants was required to be obtained before the start of the study. Study participation was voluntary. The main targets were hematologists and physicians in related fields, who may have potentially seen patients with PNH clones in their clinical practice. The two main investigators of the study screened websites of hospitals and general practices in Switzerland to identify physicians to contact. In total, 264 physicians working in Switzerland were contacted via email by a company that coordinated the study. This company anonymizes the results of the survey. In the invitation sent, the study investigators briefly described the topic and purpose of the survey. The online survey was powered by Alcimed who sent an automated email to participants once a week for 5 consecutive weeks, from September 2020 onward. Considering the rarity of this disease, the survey intended to collect opinions from healthcare professionals with at least minimal experience treating patients with PNH; therefore, two screener questions were included. The first screener question asked whether the participant has had the possibility to investigate PNH clones offering a bimodal yes/no answer. The second screener question inquired about the experience of the participant in investigating PNH clones. Three options were offered from which the participant could select one, including the option of never having investigated for PNH clones in a patient. If the participant answered the combination of “no” for the first screener question and that they had never investigated clones in a patient in the second screener question, the questionnaire was concluded and the participant was blocked from continuing with the questionnaire.

At the end of the survey, the participants were additionally asked whether they would have interest in participating in further discussion on the topic (bimodal answer yes/no). Alcimed generated a list of those candidates who reported interest to participate in further discussion and made it available to the investigators of this study.

### Statistical analysis

No formal hypothesis testing was performed. The study was not powered to detect statistically significant differences, and the sample size was, therefore, not pre-determined. Descriptive statistics were used to quantitatively summarize the data. Responses to questionnaires were calculated as proportions (*n*/*N* × 100 in %; with *N* representing the total number of participants who responded to the individual questions).

## Results

### Characteristics of the participating physicians

Over the study period of 5 weeks, 64 out of 264 physicians completed the questionnaire (total cohort), resulting in a response rate of 24.2%. The characteristics of the participating cohort are presented in Table 1. In summary, the cohort predominantly consisted of hematologists (81.3%), 67.2% of the participants worked at a university hospital, and 54.7% had more than 10 years of experience in their specialty. In total, 23 institutions were involved across 12 cantons in Switzerland.

**Table 1:** Characteristics of the participants.

	**Physicians (*N* = 64)**
	**% (** ***n*** **)**
**Canton in Switzerland**	
Aarau	4.7 (3)
Basel	17.2 (11)
Bellinzona	1.6 (1)
Bern	40.6 (26)
Geneva	3.1 (2)
Luzern	1.6 (1)
Lugano	1.6 (1)
Solothurn	1.6 (1)
St. Gallen	3.1 (2)
Thurgau	1.6 (1)
Vaud	10.9 (7)
Zürich	12.5 (8)
**Employment**	
University hospital	67.2 (43)
Cantonal/regional hospital	23.4 (15)
Private clinic	6.3 (4)
Private practice	3.1 (2)
**Medical speciality** ^a^	
Hematology	81.3 (52)
Internal medicine	32.8 (21)
Oncology	12.5 (8)
Hepatology	3.1 (2)
Pediatric hematology/oncology	1.6 (1)
Nephrology	0 (0)
**Experience (years)**	
Trainee (assistant)	15.6 (10)
< 10 years of clinical practice in my specialty	29.7 (19)
10–20 years of clinical practice in my specialty	31.3 (20)
>20 years of clinical practice in my specialty	23.4 (15)

^a^Several responses were possible.

### PNH clone testing and patients in which PNH clones are assessed

The screener questions did not exclude any participants. All participants indicated that they had access to flow cytometry for PNH clone testing. Most of the participants (76.6%, *n* = 49) perform PNH testing in their own institution's laboratory, while 18.8% (*n* = 12) send samples to other hospitals and 4.6% (*n* = 3) to private laboratories.

In total, 89% of the participants reported having searched for PNH clones in more than three patients, and only 11% had investigated PNH clones between 1 and 3 times in their medical career. The top four reasons selected to investigate the presence of PNH clones were: unexplained thrombosis occurring at unusual sites (93.8%, *n* = 60), unexplained hemolysis (92.2%, *n* = 59), direct Coombs-negative hemolysis (85.9%, *n* = 55), and aplastic anemia (84.4%, *n* = 54; Table 2).

**Table 2:** Patient types in which participants would recommend having their PNH clones investigated (*N* = 64).

**Rank**	**In which patient types are you looking for PNH clones in your practice?**	***n* (%)^a^**
1	All patients with unexplained thrombosis occurring at unusual sites (Budd Chiari, splanchnic thrombosis)	60 (93.8)
2	Patients with unexplained hemolysis	59 (92.2)
3	Patients with Coombs-negative hemolysis (high-serum LDH)	55 (85.9)
4	Patients with aplastic anemia	54 (84.4)
5	Patients with persistent unexplained cytopenia	47 (73.4)
6	Patients with the following type of MDS: refractory anemia or hypoplastic MDS	46 (71.9)
7	Patients with hemoglobinuria	41 (61.4)
8	Patients with Coombs-negative hemolysis (high-serum LDH), especially if associated with concurrent iron deficiency.	39 (60.9)
9	Patients with persistent unexplained anemia	35 (54.7)
10	No anemia, high-serum LDH, increase of reticulocyte.	25 (39.1)
11	Patients with persistent unexplained thrombocytopenia	20 (31.3)
12	Patients with unexplained iron deficiency	19 (29.7)
13	All patients with MDS	10 (15.6)

LDH, lactate dehydrogenase; MDS, myelodysplastic syndromes; PNH, paroxysmal nocturnal hemoglobinuria.

^a^Proportion of participants reporting each patient type within the total cohort (N = 64). Participants responded through a multiple-choice question and could select more than one patient type.

**Figure 1:**
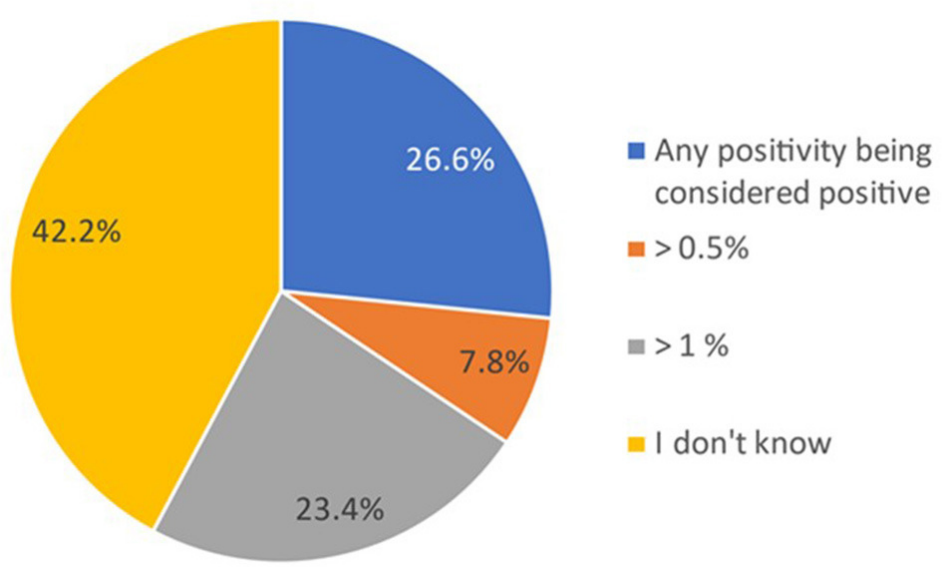
Proportion of participants indicating the cutoff used to consider a PNH clone as positive. Data in the figure are presented as the proportion of participants reporting the cutoff used for considering a PNH clone as positive within the total cohort (*N* = 64). Participants gave their responses through a multiple-choice question and could select one option.

In total, 42% (*n* = 27) of the participants did not know the cutoff value used by the laboratory to report the PNH clone as positive for the diagnosis of PNH (Figure 1).

### Follow-up of PNH clones

In patients with AA/MDS without the PNH clone at diagnosis, 60.9% (*n* = 39/64) of the physicians would continue to assess the PNH clone during follow-up.

Over half of the participants (60.9%, *n* = 39) indicated that they had patients in which a PNH clone was identified in follow-up (Supplementary Figure 1A), and 10% reported having more than 10 patients in follow-up (Supplementary Figure 1B). However, most physicians indicated having between 1 and 5 such patients (84.6%, *n* = 33; Supplementary Figure 1B). Slightly over half of the physicians (57.8%, *n* = 37) indicated that AA would be the most important reason for continued monitoring for PNH clone, followed by patients with a florid symptomatic hemolytic form of PNH (43.8%, *n* = 28) and patients with thrombosis harboring PNH clone (39.1%, *n* = 25; Supplementary Table 1). Most participants would monitor PNH clones annually (74.4%, *n* = 29), while the others would check more frequently (14.1%, *n* = 9; every 3–6 months) or never (1.6%, *n* = 1; Supplementary Figure 2).

### Management of thrombosis in a patient with PNH clone: case example I

A hypothetical case was presented involving a 35-year-old man with an unprovoked cerebral sinus thrombosis. Investigations revealed a PNH clone of 25% and an elevated LDH of 1.6 > upper limit normal (ULN), without associated marrow failure. Participants were asked about the management of thrombosis; the selection of multiple answers was possible. For this case example, eculizumab was indicated to be the most common (70.3%, *n* = 45) first treatment choice, followed by vitamin K antagonists (60.9%, *n* = 39) and heparin (unfractionated heparin, UFH, or low-molecular-weight heparins, LMWH; 53.1%, *n* = 34; Table 3).

**Table 3:** Treatment choice of participants to manage signs and symptoms in a patient with thrombosis (case example I) and to manage risks associated with a growing PNH clone in a patient with aplastic anemia (case example II).

**Treatment**	**Case example I**	**Case example II**
	A 35-year-old man was diagnosed with an unprovoked cerebral sinus venous thrombosis. He presented with mild anemia and severe fatigue while going through a very stressful moment in his life. During the work up, a PNH clone of 25% was detected. The patient does not have an associated bone marrow disorder; his current LDH level is 1.6 ULN (upper limit normal).	A 51-year-old woman was diagnosed 3 years ago with moderate aplastic anemia and a PNH clone of 9%. Under treatment with cyclosporin, her current CBC values are stable, LDH level is now 1.3 ULN (upper limit normal), and the PNH clone grows to 50%. She complains about fatigue.
		**Due to the presence of a larger PNH clone, does this patient need an additional therapeutic intervention?**
		***n*** **(%)**^**a**^
		Yes 41 (64.1)
		No 23 (35.9)
	**How would you manage the thrombosis of this patient (*****N*** **=** **64)?**	**If yes, how would you manage the risk associated with PNH for this patient (*****N*** **=** **41)?**
	***n*** **(%)**^**a**^	***n*** **(%)**^**a**^
Eculizumab	45 (70.3)	29 (64.4)
Vitamin K antagonists	39 (60.9)	14 (31.1)
Heparin/LMWH	34 (53.1)	9 (20.0)
Direct factor Xa inhibitors	13 (20.3)	9 (20.0)
Ravulizumab	11 (17.2)	8 (17.8)
Direct Thrombin inhibitors	6 (9.4)	3 (6.7)
No treatment	1 (1.6)	N/A
Aspirin	0 (0.0)	1 (2.2)

LMWH, low-molecular-weight heparin.

^a^Participants responded through a multiple-choice question and could select more than one treatment option.

### Additional therapeutic considerations in a patient with a PNH clone: case example II

A second hypothetical case was presented involving a 51-year-old woman with moderate AA and a PNH clone of 9%. She was treated with cyclosporine. Three years after this diagnosis, she complains about fatigue, her complete blood counts (CBC) remained stable, her LDH level was 1.3 >ULN, and the PNH clone had grown to 50%. The participants were asked about the need for additional therapeutic intervention due to the presence of a larger PNH clone. In total, 41 (64.1%) responded that this patient would need an additional therapeutic intervention (Table 3). Among the seven different therapeutic options offered to treat this patient, eculizumab was selected as the first additional treatment of choice (64.4%, *n* = 29), followed by vitamin K antagonists (31.1%, *n* = 14; Table 3).

### Treatment with eculizumab/ravulizumab

The questionnaire included a specific question to identify clinical situations in which the participants would consider initiating treatment with C5 inhibitors (eculizumab/ravulizumab); the selection of multiple options was possible. The two leading clinical indications were thrombosis related to PNH (93.8%, *n* = 60), followed by high LDH levels ≥1.5 × ULN and complications (67.2%, *n* = 43; Table 4).

**Table 4:** Participants indicated patient types for which they consider treatment with eculizumab/ravulizumab.

**Rank**	**When would you consider treating with eculizumab/ ravulizumab in a patient with a PNH clone?**	***n* (%)^a^**
1	Thrombosis related to PNH	60 (93.8)
2	High LDH ≥ 1.5 ULN and complications	43 (67.2)
3	High LDH ≥ 1.5 ULN and anemia (Hb < 90 g/L)	42 (65.6)
4	High LDH ≥ 1.5 ULN and symptoms	37 (57.8)
5	Pregnancy (and for at least 3 months postpartum)^b^	28 (43.8)
6	History of thrombosis, and now PNH clone	23 (35.9)
7	PNH clones ≥ 10%	9 (14.4)
8	High LDH ≥ 1.5 ULN only	4 (6.3)

Hb, hemoglobin; LDH, lactate dehydrogenase; ULN, upper limit normal; PNH, Paroxysmal nocturnal hemoglobinuria.

^a^Proportion of participants reporting clinical situation options within the total cohort (N = 64). Participants responded through a multiple-choice question and could select more than one option.

^b^Only eculizumab.

### Tests/tools to monitor asymptomatic patients with PNH clones

**Figure 2:**
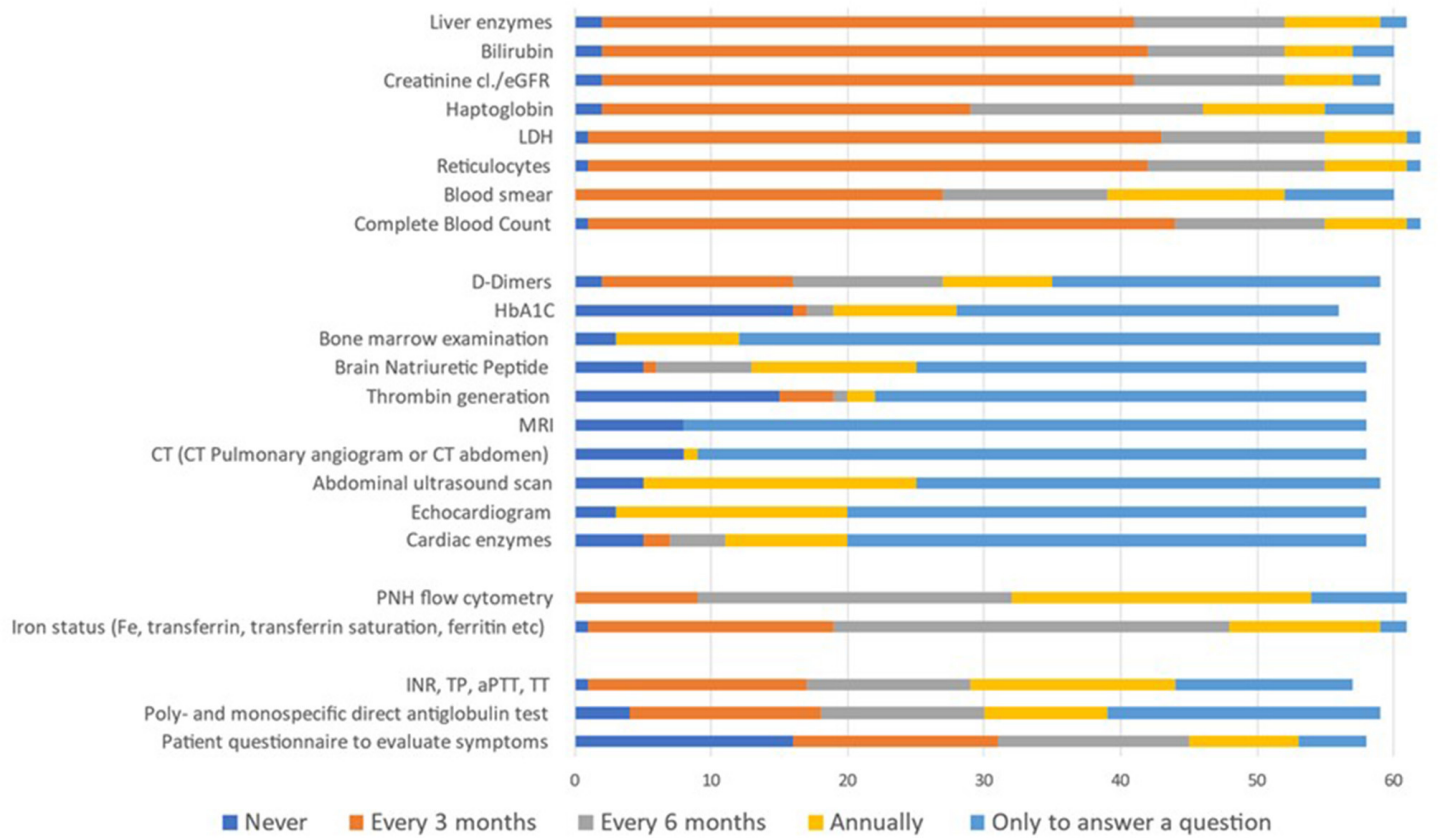
Type of tests/tools and frequency used to monitor asymptomatic patients with PNH clones. Data in the figure are presented as the proportion of participants reporting each test/tool option within the total cohort (*N* = 64). Participants indicated test/tool options through a multiple-choice question and could select more than one option.

The last question aimed to identify what laboratory tests/tools participants commonly used to monitor asymptomatic patients with PNH clones. A list of 23 test/tool options was presented. Participants could indicate the frequency with which they would use each of them (never, every 3 or 6 months, annually, or only to answer a specific clinical question; Figure 2). Complete blood count (CBC, 69.4%), reticulocytes (66.1%), LDH (67.7%), creatinine (cl./eGFR; 66.1%), bilirubin (66.7%), and liver enzymes (63.9%) constituted the commonly and most frequently (every 3 months) used tests/tools for monitoring. The reported use of haptoglobin, PNH flow cytometry, and hemostasis parameter was very scattered. Furthermore, 47% of participants agreed that bone marrow investigation, cardiac enzymes, echocardiogram, abdominal ultrasound, CT scan (pulmonary angiogram or abdomen), magnetic resonance imaging (MRI), thrombin generation, and brain natriuretic peptide (BNP) are justified to answer a specific clinical question.

At the end of the survey, the participants were asked whether they would have interest in participating in further discussions on the topic. Of the 64 participants, 37 (57.8%) expressed their interest, and, in the end, 14 participated in a panel representing different Swiss centers. Some of the panel members had vast experience in the field. The panel joined in a first online meeting on 1 March 2021, where the results of the survey were presented. The panel worked on the interpretation and discussion of the results of the survey, which is integrated into this manuscript.

## Discussion

PNH is a rare, life-threatening, chronic hematologic disease caused by uncontrolled terminal complement activation. In Switzerland, 56 patients diagnosed with PNH have been included in the Swiss PNH Registry, since its start in 2012 (data as of 2021) (29). In addition to its rarity, the major challenge of PNH is achieving an early diagnosis, which is often hindered by its clinically heterogeneous presentation through a variety of non-specific symptoms. International guidelines on screening, diagnosis, and management of PNH have been developed to assist clinicians (6, 31–35). Yet, PNH often remains misdiagnosed (36), and therefore, real-world data on clinical practices and experiences with PNH are important for finding ways to improve early diagnosis of this rare disease. In our study conducted in Switzerland, we could confirm the interest among hematologists and other physicians in this topic through a response rate of 24%. Previously published surveys on PNH focused primarily on specific topics such as the use of eculizumab in pregnancy or laboratory methodologies to assess PNH clones within the United Kingdom National External Quality Assessment Service (UK NEQAS) (30, 37). To the best of our knowledge, there are no published surveys collecting individual opinions from physicians. The location of the participating hematologists/physicians across Switzerland included all nine designated centers for rare diseases in Switzerland (38). The number of participating hematologists (81%, *N* = 52) out of all participant physicians (*N* = 64) represents 23% proportion of the hematologists in Switzerland [*n* = registered 224 hematologists in 2021 reported 23.03.2022; source FMH-Ärztestatistik/Statistique médicale de la FMH (39)].

In our survey, unexplained thrombosis in unusual sites was ranked first among clinical situations in which PNH clones must be investigated. Despite the relevance of thrombosis as a complication in PNH, it is necessary to emphasize that in a large study of 3,939 peripheral blood samples for diagnostic screening of PNH by flow cytometry, only a minor fraction of patients tested for unexplained thrombosis in the absence of cytopenia had a PNH clone (0.4%) (40). The diagnosis of PNH should be considered in the presence of unexplained thrombosis, both venous and arterial. Thrombosis in unusual venous sites (hepatic, mesenteric, cerebral, and dermal veins) leads to severe morbidity and is a common cause of mortality in PNH (41, 42), requiring urgent treatment. Patients with PNH-related thrombosis usually have larger PNH clones (>50%) (43), and clone size has been observed to correlate with the severity of the disease (44, 45), but it is certainly important to note that thrombosis can occur with clones of size below 50%.

Unexplained hemolysis and Coombs-negative hemolysis (high-serum LDH) were ranked second and third, respectively. Intravascular hemolysis is the primary clinical finding associated with PNH (42, 43, 45, 46) and is associated with highly increased thrombotic risk (47). Indeed, isolated hemolysis has a 19% diagnostic yield, therefore, in line with the collected answers in this survey (40). Now, it is known that direct Coombs positive hemolytic conditions may also conceal the presence of PNH clones (21). Except for a few rare cases (25), PNH clone size correlates closely with hemolysis and LDH levels. Hence, LDH ≥1.5 ULN is a risk factor for patients with PNH clones (48).

AA was ranked fourth (out of 13) as a patient category in which PNH clones should be investigated. Actually, this is the pathology most frequently associated with PNH clones. The highest frequency of PNH-positive cases is observed among patients screened because of bone marrow failure syndromes with a frequency of 33%, particularly among those with AA with a 45% diagnostic yield (40). The answers collected in the survey may be due to an overestimation of the hemolytic forms by the participants to answer the questions. Compared to patients with “classical PNH,” smaller-sized clones can be found in patients with AA (48). Moreover, PNH frequently occurs during the clinical course of AA (49), and a proportion of patients with AA may develop PNH resulting from late hematological complications or in the course of immune-suppressive treatment (50). PNH clones can also be detected in patients with MDS, but less frequently than in patients with AA (28), and this was also ranked lower in our survey.

In this survey, all participants confirmed accessibility to PNH clone determination by flow cytometry. Interpreting the flow cytometry results for PNH clones is an important step for screening, diagnosing, and monitoring patients with known or suspected PNH (43). In our survey, knowledge regarding the cutoff used in the report of flow cytometry to consider the presence of a PNH clone was diverse: 42% of the participants had no knowledge of the cutoff. PNH clones are detectable in only a fraction of patients at risk, and the methodology can be complex and expensive. Previous studies showed that the risk of misdiagnosing PNH by flow cytometry is higher among less experienced laboratories, particularly for those samples presenting with a low number of GPI-deficient cells (7, 40). Some models of PNH screening regional programs that standardized and simplified the methodology and implemented diagnosis confirmation by introducing a reference center are the models to follow in order to optimize the diagnostic difficulties observed in this survey (51). The most critical and controversial aspects of PNH diagnosis, reporting, and proper terminology have already been established in authoritative articles (31, 52). Since flow cytometry is currently the “gold standard” for laboratory diagnosis of PNH, our findings strongly suggest that laboratories in Switzerland need to adhere to international guidelines regarding the assessment and reporting of PNH clone results.

Patients with established diagnoses of hemolytic PNH or PNH in the setting of an underlying bone marrow disease (e.g., AA, or hypoplastic MDS) should be regularly assessed for clone size. According to the British guidelines, AA patients should be screened for PNH at the time of AA diagnosis. If the test is persistently negative, it should be done every 6 months for 2 years before moving to annual testing until symptoms/signs develop. If the PNH screen is, or becomes, positive, the test should be done every 3 months for the first 2 years, with the frequency reduced only if the proportion of PNH cells remains stable (3). Patients under treatment with complement inhibitors (1) and during the early phase after allogeneic stem cell transplantation need a follow-up clone size assessment. In our survey, only 61% of the participants mentioned checking for the existence of a PNH clone during follow-up in AA/MDS patients where a clone was not detected at diagnosis. This could be explained by the lack of clinical experience of those who participated in the survey. Most of the participants suggested checking annually (74%), which is correct if the disease is stable (6).

Thrombosis, which sometimes can be in atypical sites such as abdominal veins and cerebral sinuses, is a cardinal manifestation of PNH, and a leading cause of morbidity and mortality (1, 41, 53). Terminal complement inhibition is the only proven therapy preventing thrombosis (recurrence) in patients with PNH (1, 54–57). Management of thrombosis in a patient with a PNH clone was the first hypothetical case included in our survey. Most participants were selected for therapy with C5 inhibitors with 70.3% considering eculizumab and fewer (17.2%) ravulizumab. We point out here that, at the time of the survey, ravulizumab had been available in Switzerland for < 6 months.

In addition to the prevention of complement-mediated intravascular hemolysis, acute thrombosis should be managed with anticoagulation similar to patients without PNH (2, 53). Most guidelines recommend starting with unfractionated heparin/low-molecular-weight heparin (LMWH) and transitioning to vitamin K antagonist (VKA). Those were consistently the two preferred answers in the survey with 53.1, and 60.9%, respectively. Since PNH is a rare disease, probably not many studies will be started to test for safety and efficacy of direct oral anticoagulants (DOACs) in PNH patients. To the best of our knowledge, available data are limited to one case report and one small case series, both reporting rivaroxaban use (58, 59). Most experts in the field would only consider DOACs an option in the setting of secondary venous thromboembolism VTE prevention in patients with controlled disease on terminal complement inhibition (60). However, direct factor Xa inhibitors and direct thrombin inhibitors were chosen by 20.3 and 9.4% of the participants, respectively. This might represent the general liberal use of DOACs for other indications in Switzerland and the use here “by analogy.”

Discontinuation of anticoagulation within the context of well-controlled hemolysis in treated PNH is a controversial issue as well. The consensus is to maintain anticoagulation for a minimum of 3–6 months. Given the strong thrombotic potential of complement-mediated hemolysis, it remains debatable whether long-term anticoagulation is still needed while the patient is under complement-inhibitory therapy. It might be appropriate to discontinue anticoagulation in patients with stable disease under terminal complement inhibition (60, 61). In PNH patients, who are not treated with complement inhibitor, the cumulative risk of recurrent thrombosis is high and proportional to PNH-clone size (55, 56).

The hypercoagulable state in PNH is multifactorial. The contribution of platelets derived from the mutant stem cell clone susceptible to complement-mediated activation plays an important role in the pathogenesis of thrombophilia in this situation (62); however, clinical data demonstrating a benefit of anti-aggregation in PNH are lacking, and in line with this, none of the participants considered aspirin as a valid therapeutic option.

The second case presented in the survey meets the criteria for moderate acquired AA but due to the growing PNH clone is at high risk for thrombosis. Thrombosis may occur in any PNH patient, but those with a PNH clone >50% are at greatest risk (63). Thus, in this case, additional therapeutic intervention is needed. The clone size *per se* is not an indication to start a complement-inhibitor therapy. The classic recommendations require overt hemolysis symptoms and/or and transfusion-dependence, PNH-related thrombosis, complications associated with hemolysis (e.g., renal failure), pregnancy in active PNH (and for at least at 3 months postpartum), and symptomatic hemolytic anemia (LDH >1.5 × ULN). Exceptionally, the therapy can be considered in cases that do not fulfill these criteria, but present clinical conditions that justify its use (32, 33, 64, 65). The case presented in the survey did not formally meet any of the criteria listed above. Therefore, the high rate of responses in favor of initiating complement inhibitors treatment from the participants for this case is surprising. Indeed, in patients with large clones exceeding 50% size, thrombosis prophylaxis or full anticoagulation depending on the estimated risk should be recommended. Identifying high-risk PNH patients in need of therapy is lifesaving. In patients with PNH, the main cause of death is the complication of venous thrombosis (34, 42). Venous thrombosis and intravascular hemolysis have been proven to decrease when patients were treated with eculizumab (Alexion Pharmaceuticals, Inc.), a humanized monoclonal antibody that inhibits terminal complement activation (66). Most of the participants (94%) in our survey acknowledged thrombosis related to PNH as the most important clinical situation requiring treatment with complement inhibitors.

The clinical situation of LDH ≥ 1.5 ULN in combination with complications was identified as the second important indication to treat patients with PNH. LDH is an indicator of intravascular hemolytic activity (67) and is considered the best measure for the severity of disease in the vast majority of patients (45). According to data published by the Korean Registry (68), a high LDH reflects high disease activity and an increased risk of thrombosis, renal impairment, pulmonary hypertension, and death.

The observation that eculizumab was predominantly chosen over ravulizumab can be explained by the fact that ravulizumab was approved in Switzerland in early 2020, which is the same year during which the survey was carried out. Since the introduction of ravulizumab in Switzerland, its adoption is increasing to treat patients who are naive to eculizumab treatment and for patients who are clinically stable on eculizumab (29). Hence, ravulizumab is likely to replace eculizumab as the standard of care to treat PNH, which confirms observations elsewhere (69).

The results of this survey align with recommendations for having CBC, reticulocytes, LDH, creatinine, bilirubin, and liver enzymes assessed every 3 months for monitoring asymptomatic patients with PNH clones (6, 43). Bone marrow evaluation is not standard in the investigation of all PNH patients; the indication is restricted to those cases with additional cytopenia or when an underlying associated disease such as AA, MDS, or other is suspected (43). During the follow-up of the PNH, this procedure will be performed only to answer specific diagnostic questions.

This survey has strengths and limitations. This survey collected information for the first time in Switzerland on the daily practices for the recognition and diagnosis of this rare disease. It also brings together opinions about the clinical approach to situations in patients with PNH that are not well-defined in international guidelines. It made it possible to bring together participants from different institutions to evaluate and discuss the results and thus identifying the unmet needs for better awareness of PNH in the country. Among the limitations, greater participation of the canton of Bern was observed. This could be due to the methodology used to contact the participants, in which the company coordinating the survey (Alcimed) contacted potential participants by sending emails signed by the two main researchers of the survey, both of whom work in the canton of Bern, which may have provided additional motivation for some local colleagues to participate. This greater representativeness could have influenced the results of this survey, being a potential limitation of the study.

## Conclusion

Considering that PNH is a rare disease, the degree of participation in the survey shows interest from the contacted medical community. The results demonstrate the awareness of the participants regarding the association of thrombosis, hemolysis, and cytopenia with a PNH clone. While easy accessibility to flow cytometry for the investigation of PNH clones was reported, technical aspects such as the definition of a small clone seem to be less known. Our results point to a need for adopting guidelines regarding the assessment, reporting, and interpretation of the PNH clone results among the laboratories in Switzerland. Even though, our results confirm the general rule about monitoring clone size annually in stable disease, there is a need to stratify patients according to the risk for appropriate follow-up. The answers in the clinical cases were scattered. Despite the existence of several international guidelines, clear information on topics such as the type of anticoagulant to use and its duration, as well as the indication for treatment with complement inhibitors in some borderline situations, is needed. The analysis and the discussion of this survey represent the basis for understanding the unmet needs of PNH clone assessment and clinical practice in Switzerland.

## Data availability statement

The original contributions presented in the study are included in the article/Supplementary material, further inquiries can be directed to the corresponding author.

## Author contributions

AR and SZ designed the manuscript and prepared the used questionnaire. Alcimed, AR, and HD did the data analysis. AR and HD wrote the first draft. All authors participated in the meeting where the data were presented and discussed, performed bibliographic searches to substantiate what was discussed for each question, contributed to the data discussion, revised the manuscript, and approved the final draft.
