# Supplementary material for: Swiss Survey on current practices and opinions on clinical constellations triggering the search for PNH clones
Source: Front Med (Lausanne). 2023 Jul 26;10:1200431. doi: 10.3389/fmed.2023.1200431 (PMC10410560; doi:10.3389/fmed.2023.1200431)
Supplement: Supplementary file 1 [file Data_Sheet_1.PDF]

Survey on PNH Clone

Dear Colleagues,

PNH (Paroxysmal Nocturnal Hemoglobinuria) is a rare acquired hematopoietic stem cell disorder, classically characterized by complement mediated hemolytic anemia and hemoglobinuria with a broad constellation of clinical findings.

Diagnostic testing has evolved significantly, the determination of PNH clones using highly sensitive flow cytometry has led to the identification of a growing pool of patients with PNH clones.

We know that small to moderate PNH clone are frequently found in patients with acquired aplastic anemia and in some forms of myelodysplastic syndromes.

Moreover, PNH clones can be found in patients with unexplained asymptomatic cytopenia or in patients with thrombosis in unusual site.

Thrombosis is one of the most harmful complications of the presence of PNH clones. It is hard to predict PNH complications and thrombosis in such patients

This is a national survey; we would like to better understand some aspects related to your current practice and get your opinion on clinical constellations that trigger the search for a PNH clones. In addition, questions related to your access to investigate PNH clones in your patients and how do you monitoring patients in whom a PNH clone has been diagnosed are some of the objectives to evaluate in this survey.

The aggregate results obtained in this survey will be discussed with a PNH panel of experts in order to prepare a proposal to be presented at a specialized meeting on the subject.

Please take a few minutes to answer the following questions, thank you.

Demographic questions (1 to 3)

1. **What is your medical specialty?** *please select all relevant options*
    a. Hematology
    b. Oncology
    c. Hepatology
    d. Internal Medicine
    e. Nephrology
    f. Other

2. **What of the following describes closely the length of your professional career:**
    a. Trainee (Assistant)
    b. < 10 years of clinical practice in my specialty
    c. 10 to 20 years of clinical practice in my specialty
    d. >20 years of clinical practice in my specialty

3. **What of the following describes closely the place where you currently work:**
    a. University hospital
    b. Cantonal/Regional hospital
    c. Private clinic
    d. Private practice
    e. other

Two questions to decide whether the participant is our target:

4. Do you have access to flow cytometry testing of PNH clones in your patients?

a) yes

b) no

if yes: (open new window) *please select only one options*

☐ the test is performed in the laboratory of the institution where I work

☐ we send samples to another hospital

☐ we send samples to a private laboratory

5. Please, select one of the following options that suit best with your own experience searching for PNH clones in your patients.

☐ A. I have never had investigated PNH-Clones in my patients.

☐ B. I have investigated PNH-clones in my patients only exceptionally (less than 3 times in my medical career)

☐ C. I have investigated PNH-Clones in many cases (more than 3 patients at least).

6. In which patient types are you looking for PNH clones in your practice?
Please select all relevant options

☐ Patients with hemoglobinuria

☐ Patients with unexplained hemolysis

☐ Patients with Coombs-negative hemolysis (high serum LDH).

☐ Patients with Coombs-negative hemolysis (high serum LDH), especially if associated with concurrent iron deficiency.

☐ No Anemia, high serum LDH, increase of reticulocyte.

- ☐ All patients with unexplained thrombosis occurring at unusual sites (Budd Chiari, splanchnic thrombosis)
- ☐ Patients with Aplastic Anemia
- ☐ All patients with MDS
- ☐ Patients with the following type of MDS: refractory anemia or hypoplastic MDS
- ☐ Patients with persistent unexplained cytopenia
- ☐ Patients with persistent unexplained anemia
- ☐ Patients with persistent unexplained thrombocytopenia

7. In AA/MDS patients without a PNH clone at diagnosis, do you check again the existence of a PNH clone during Follow-up?

- ☐ yes
- ☐ no

If yes, how often do you check?

- ☐ Every 3 months
- ☐ Every 6 months
- ☐ Every year
- ☐ Never

8. Do you have patients in follow-up with PNH clone?

a. yes

b. no

10. How many patients with PNH clones do you have in follow-up?

a. □ 0

b. □ 1-2

c. □ 2-5

d. □ 5-10

e. ☐ >10

11. **Patients with PNH clones you follow in your practice are more likely to be:**

Please select all relevant options

a. AA patients
b. PNH patients with florid symptomatic form related to hemolysis
c. PNH patients without hemolysis
d. MDS patients
e. Patients harboring PNH without a clear diagnosis
f. Patients harboring a PNH clone suffering thrombosis
g. Autoimmune diseases

12. In the report of flow cytometry you receive for your patients, do you know which is the cut-off used to consider a PNH clone as positive.

a. Any positivity would be consider positive.
b. > 0.5%
c. > 1%
d. I do not know

13. Case example: 35-year old male patient was diagnosed with an unprovoked cerebral sinus venous thrombosis. He presented with mild anemia and severe fatigue while going through a very stressful moment in his life. During the work up, a PNH clone of 25% was detected. The patient does not have an associated bone marrow disorder; his current LDH level is 1.6 ULN (upper limit normal).

How would you manage the thrombosis of this patient? *Tick all appropriate boxes*

☐ No treatment

☐ Vitamin K antagonists

☐ Heparin /LMWH

☐ Direct factor Xa inhibitors

☐ Direct Thrombin inhibitors

☐ Aspirin

☐ eculizumab

☐ ravulizumab

14. PNH clones in patients <u>with</u> AA/MDS without hemolysis: Case example: female 51-year old patient, 3 years ago was diagnosed with moderate AA and a PNH clone of 9%. Under treatment with cyclosporin,

her current CBC values are stable, LDH level is now 1.3 ULN, the PNH clone grows to 50%. She complains about fatigue.

14. a. Due to the presence of a larger PNH clone, does this patient need an additional therapeutic intervention?

c. Yes
d. No

14. b. How would you manage the risk associated with PNH for this patient? *Tick all appropriate boxes*

- ☐ No treatment
- ☐ Vitamin K antagonists
- ☐ Heparin /LMWH
- ☐ Direct factor Xa inhibitors
- ☐ Direct Thrombin inhibitors
- ☐ Aspirin
- ☐ eculizumab
- ☐ ravulizumab

15. When would you consider treating with eculizumab/ ravulizumab in a patient with a PNH-Clone: *Tick all appropriate boxes.*

- ☐ PNH clones ≥ 10%
- ☐ High LDH ≥1.5 ULN only
- ☐ High LDH ≥1.5 ULN and symptoms
- ☐ High LDH ≥1.5 ULN and complications
- ☐ High LDH ≥1.5 ULN and Anemia (Hb <90g/L)
- ☐ Thrombosis related to PNH
- ☐ History of thrombosis, and now PNH clone.
- ☐ Pregnancy (and for at least 3 months post-partum) (only Eculizumab)

16. What are the tests/tools that you are using to monitor your asymptomatic patients with PNH clones? *For each item checked, please indicate the frequency of testing*

Test	Never	Every 3 months	Every 6 months	Annually	Only to answer a question
Patient questionnaire to evaluate symptoms					
Complete Blood Count					
Blood smear					
Poly- and monospecific direct antiglobulin test					
Reticulocytes					
LDH					
Haptoglobin					
PNH flow cytometry					
Bone marrow examination					
HbA1C					
INR, TP, aPTT, TT					
D-Dimers					
Thrombin generation					
Creatinine cl./eGFR					
Bilirubin					
Liver enzymes					
Iron status (Fe, transferrin, transferrin saturation, ferritin etc)					
Brain Natriuretic Peptide					
Cardiac enzymes					
Echocardiogram					
Abdominal ultrasound scan					
CT (CT Pulmonary angiogram or CT abdomen)					
MRI					
