# Supplementary material for: Swiss Survey on current practices and opinions on clinical constellations triggering the search for PNH clones
Source: Front Med (Lausanne). 2023 Jul 26;10:1200431. doi: 10.3389/fmed.2023.1200431 (PMC10410560; doi:10.3389/fmed.2023.1200431)
Supplement: Supplementary file 2 [file Data_Sheet_2.docx]

**Swiss Survey on current practices and opinions on clinical constellations triggering the search for PNH clones**

Alicia Rovó^a^, Mathilde Gavillet^b^, Beatrice Drexler^c^, Peter Keller^d^, Jenny Sarah Schneider^a^, Giuseppe Colucci^e^, Yan Beauverd^f^, Hendrika Anette van Dorland^g^, Matthias Pollak^a^, Adrian Schmidt^h^, Andrea De Gottardi^i^, Marina Bissig^j^, Thomas Lehmann^k^, Michel A. Duchosal^b^ and Sacha Zeerleder^l^

^a^ Inselspital, Bern University Hospital, Department of Hematology and Central Hematology Laboratory, Bern

^b^ Service and Central Laboratory of Hematology, Department of Oncology and Department of Laboratory Medicine and Pathology, Lausanne University Hospital (CHUV), Lausanne

^c^ Division of Hematology, University Hospital Basel, Basel

^d^ Hospital Langenthal, Langenthal

^e^ Clinica Sant’Anna, Lugano

^f^ Division of Hematology, Geneva University Hospitals and Faculty of Medicine, Geneva

^g^ Appletree CI Group AG, Winterthur, Switzerland

^h^ Department of Internal Medicine, Clinic of Medical Oncology and Hematology, Municipal Hospital Zurich Triemli, Zurich

^i^ Servizio di Gastroenterología e Epatologia, Ente Ospedaliero Cantonale, Università della Svizzera Italiana, Lugano

^j^ Department of Medical Oncology and Hematology, University Hospital of Zurich, Zurich

^k^ Kantonsspital St. Gallen, Clinic for Medical Oncology and Hematology, St. Gallen

l Department of Hematology, Kantonsspital Luzern, Lucerne and University of Bern, Bern, Switzerland

**Corresponding Author**

Prof. Dr. med Alicia Rovó

INSELSPITAL, Bern University Hospital

Department of Hematology and Central

Hematology Laboratory

Freiburgstrasse, BHH A156

CH- 3010 Bern

Switzerland

Tel + 41 31 632 3313

Email: [alicia.rovo@insel.ch](mailto:alicia.rovo@insel.ch)

**Supplement**

**Supplement I Survey Questionnaire**

**Supplement II Supplemental Figure legends**

**Supplement II Supplemental Figures and Tables**

**Suppl. Figure legends**

**Suppl. Figure 1**

**A. Proportion of participants indicating having patients with PNH clones in follow-up**

Data in the figure are presented as the proportion of participants reporting having patients with PNH clones in follow-up within the total cohort (N = 64). Participants gave their response through a bimodal question.

**B. Number of patients with PNH clones in follow-up**

Data in the figure are presented as the proportion of participants reporting the number of patients with PNH clones in follow-up within the cohort of participants having patients with PNH clones in follow-up (N = 39). Participants gave their response through a multiple-choice question and could select one option.

**Suppl. Figure 2.**

**Frequency of checking PNH clones in patients with AA/MDS**

Data in the figure are presented as the proportion of participants reporting the number of patients with PNH clones in follow-up within the cohort of participants having patients with PNH clones in follow-up (N = 39). Participants gave their response through a multiple-choice question and could select one option.

**Supplemental Figures**

Suppl. Figure 1

1.
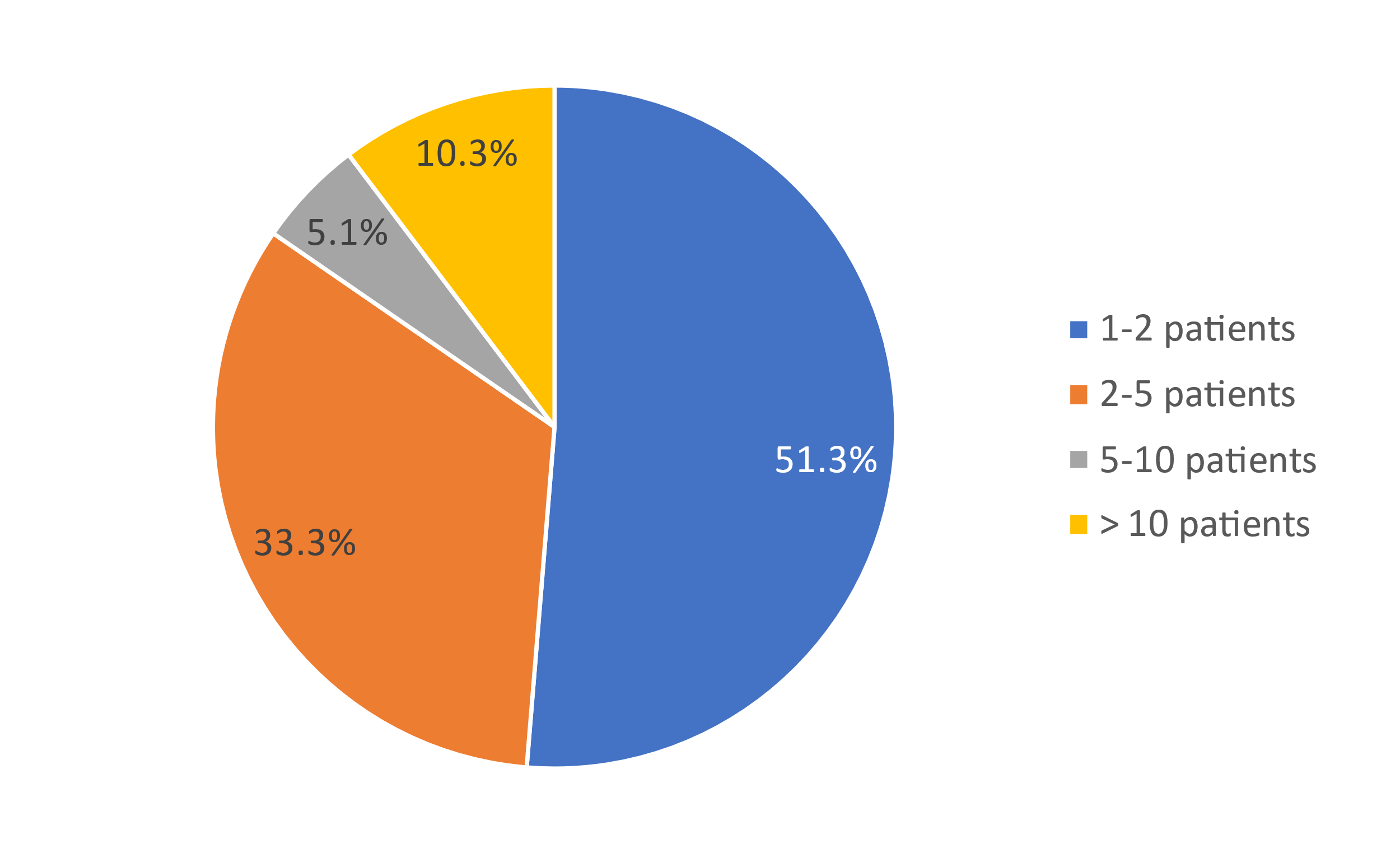

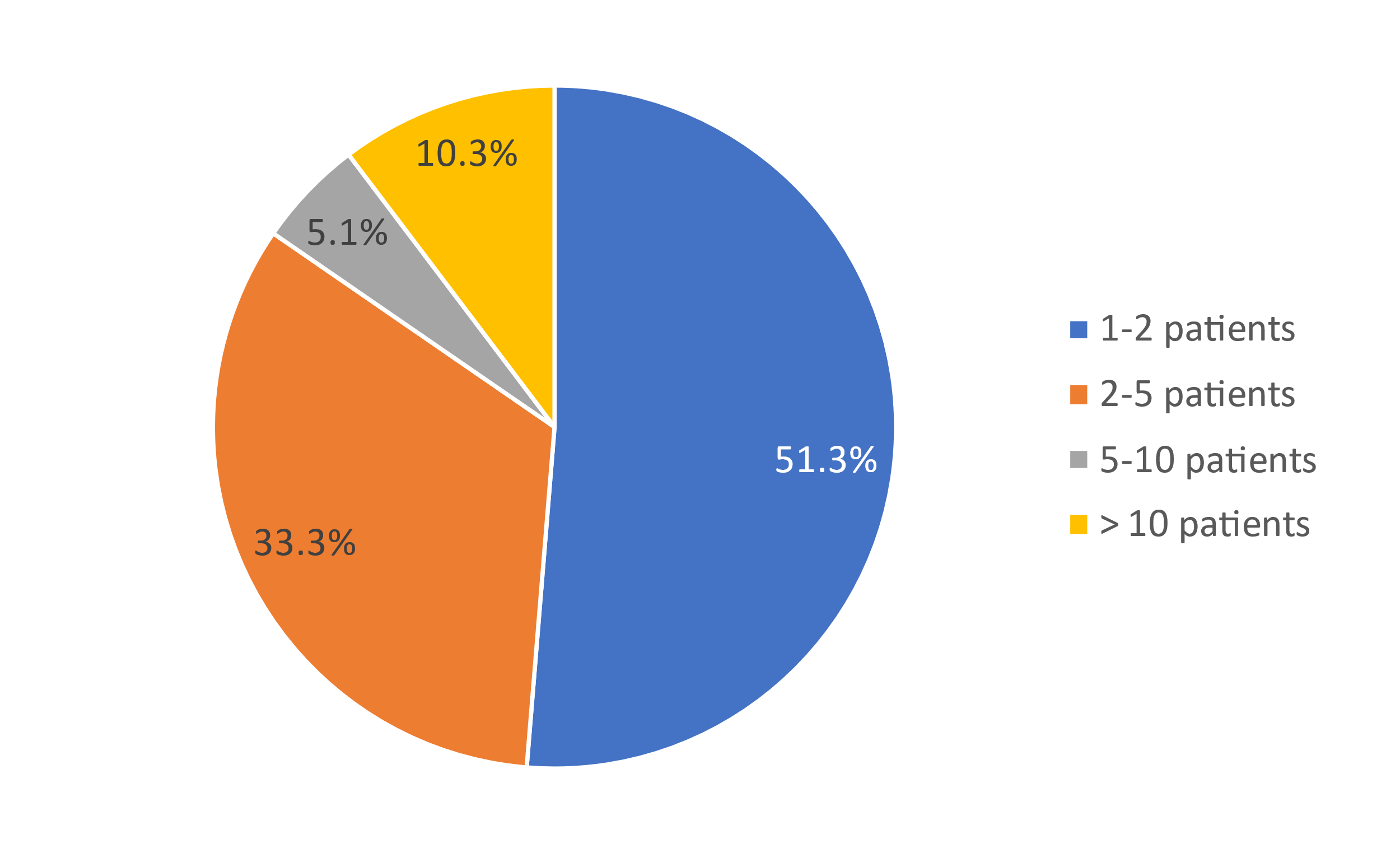

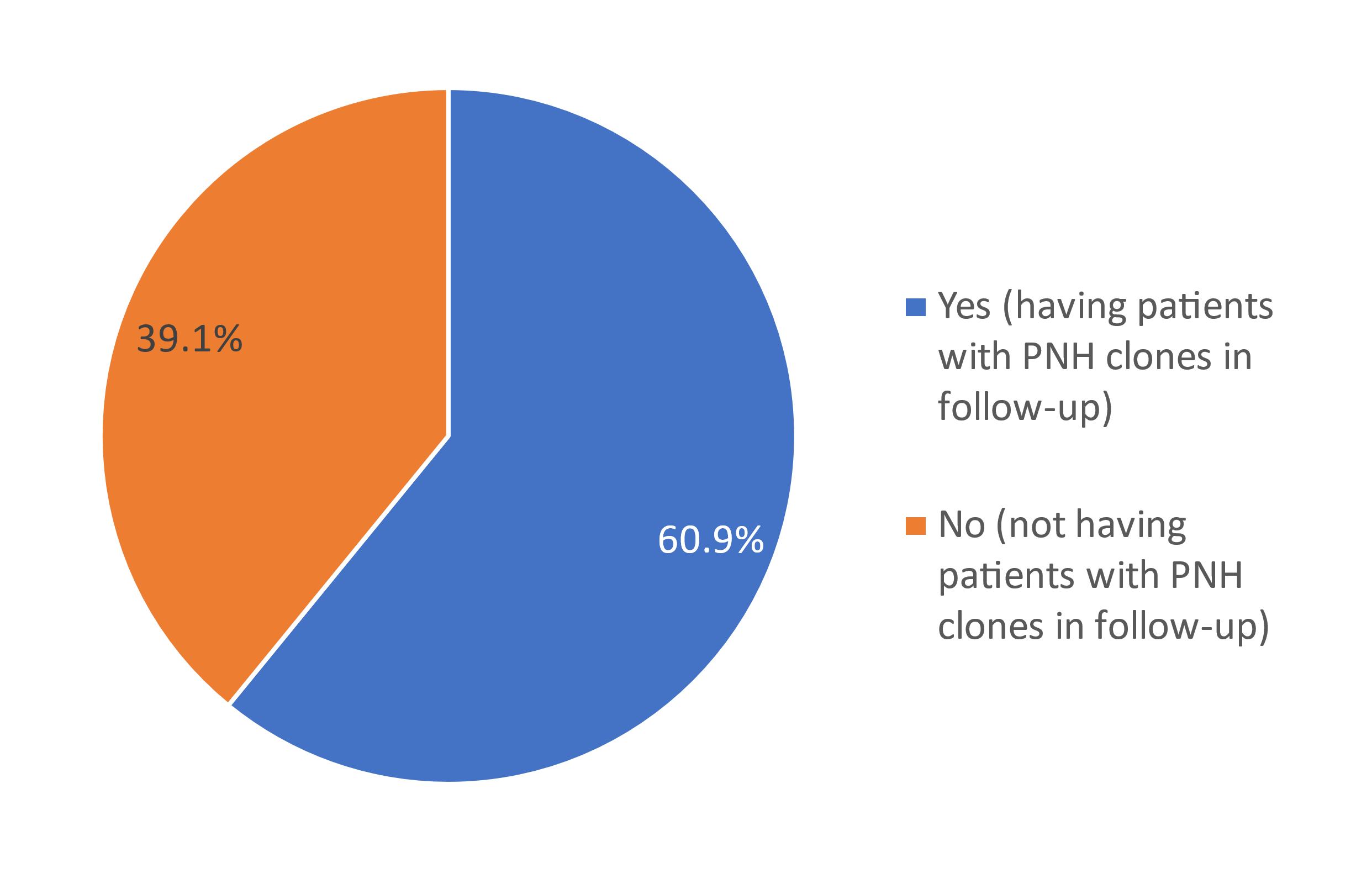
B.

Suppl. Figure 2.


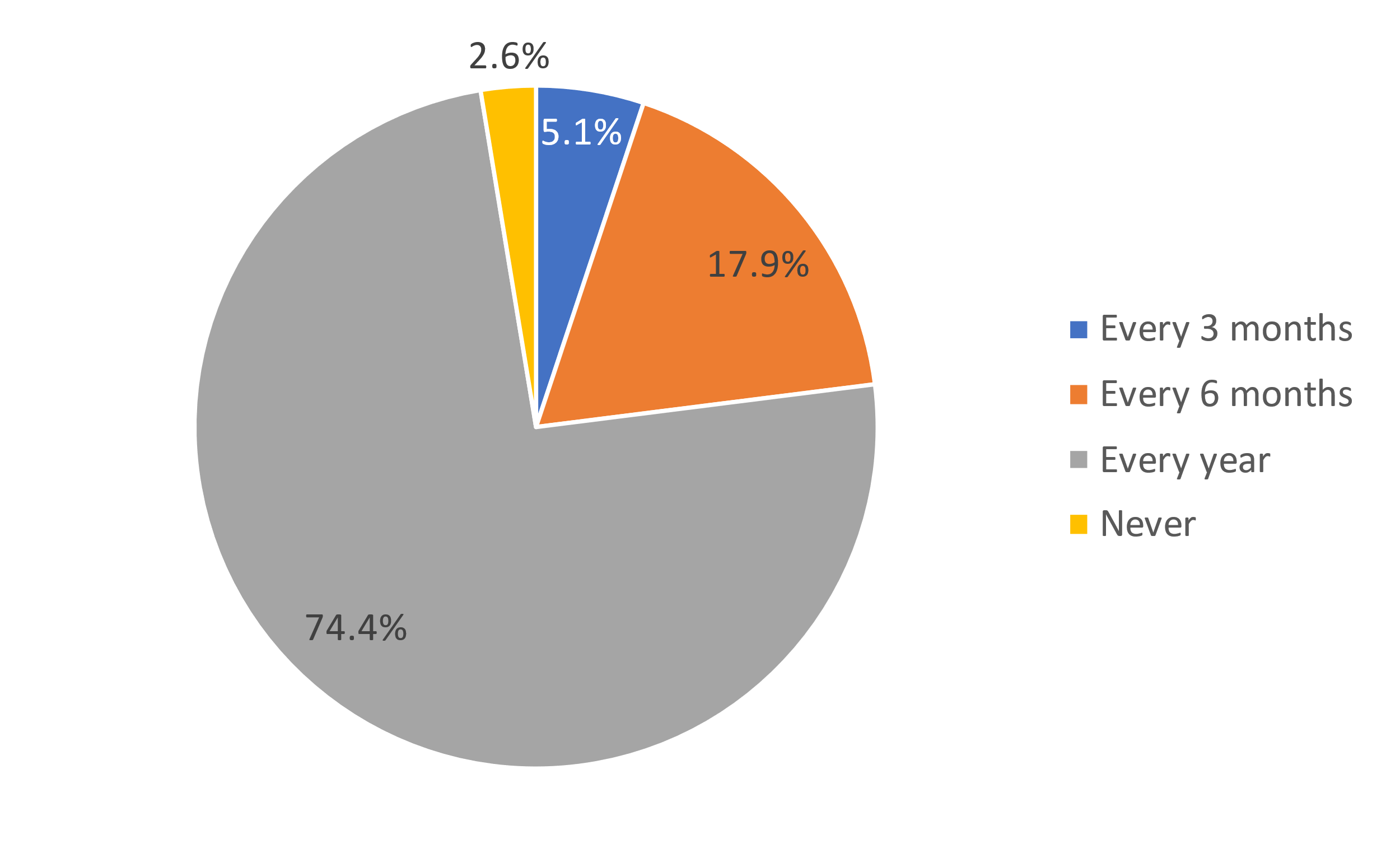


**Supplemental Tables**

**Suppl. Table 1. Participants indicating type of patients with PNH clones who are followed in their practice**

| **Rank** | **Patients with PNH clones you follow in your practice are more likely to be** | **n (%^1^)** |
| --- | --- | --- |
| 1 | Patients with aplastic anemia | 37 (57.8) |
| 2 | Patients with PNH and with florid symptomatic form related to hemolysis | 28 (43.8 |
| 3 | Patients harboring a PNH clone suffering thrombosis | 25 (39.1) |
| 4 | Patients with MDS | 21 (32.8) |
| 5 | Patients with PNH without hemolysis | 17 (26.6) |
| 6 | Patients harboring PNH without a clear diagnosis | 13 (20.3) |
| 7 | Autoimmune diseases | 3 (4.7) |

Abbreviations: MDS, myelodysplastic syndromes; PNH, paroxysmal nocturnal hemoglobinuria

^1^ Proportion of participants reporting patient types within the total cohort (N = 64). Participants responded through a multiple-choice question and could select more than one option.
